# Analysis of total phenolic contents, flavonoids, antioxidant and antibacterial activities of *Croton macrostachyus* root extracts

**DOI:** 10.1186/s13065-022-00822-0

**Published:** 2022-05-12

**Authors:** Dessie T. Ayele, M. L. Akele, A. T. Melese

**Affiliations:** Department of Chemistry, College of Natural and Computational Sciences, University of Gondar, P. O. box 196, Gonder, Ethiopia

**Keywords:** Antioxidant activity, *Croton macrostachyus*, Ethiopia, Flavonoids, Medicinal plants, Total polyphenol

## Abstract

Plants are good sources of various bioactive substances and have significant importance for the discovery of new drugs. In this study, *Croton macrostachyus* plant roots from six different sites in the Central Gondar Zone, Ethiopia, were collected; and their phenolic and flavonoid contents, as well as antioxidant and antibacterial activities were investigated. Total phenolics and flavonoid contents of the root extracts were determined by the Folin–Ciocalteu and aluminum chloride methods, respectively. The antioxidant activity was determined by the DPPH (2,2-diphenyl-1-picrylhydrazyl) free radical scavenging method. Moreover, the antibacterial activities were evaluated by disk diffusion method. Results revealed that total polyphenols and flavonoid contents were in the range of 802 ± 53–1557 ± 75 mg GAE/100 g and 342 ± 26–745 ± 32 mg CE/100 g, respectively. Root extracts of C. macrostachyus were found to have higher antioxidant activities ranging from 3.53 ± 0.38 to 6.38 ± 0.62 mg AAE/g sample. They also showed inhibition zones of 5.8, 6.2, 5.9 and 6.0 mm for *Staphylococcus aurous*, *Staphylococcus pneumonia*, *Escherichia coli* and *Klebsiella pneumonia*, and had equivalent potency with the reference Gentamicin antibiotic. Pearson correlation result indicated a strong relationship between total polyphenol contents and their respective antioxidant activities. This study articulates that the root extracts accumulated a substantial quantity of polyphenols and bears a considerable antioxidant activity.

## Introduction

Medicinal plants have high bioactive compounds [1]. These plants have nutritional and biologically active ingredients, synthesized during their growth and accumulated from the environment [2]. In spite of the enormous advances in modern medication in recent decades, plants still make a vital contribution to improve human health [3]. Regardless of the use of many plants as daily food, some of them known as medicinal plants (MP), have been used in traditional medicine (TM) as therapeutic resources as well as in changeable pharmaceutical preparations [4]. That is why, 80% people in the world utilize traditional medicine because of their improved cultural suitability, compatibility with the human body, and smaller side effects [5].

Medicinal plants are characterized as a large source of new compounds that aid for the preparation of new drugs [6]. Therapeutic activity of plants is because of their biologically active polyphenolic compounds, typically flavonoids and phenolic acids which possess antioxidant, anti-lipoxygenase and anticancer activities [7]. They have been vital sources of both preventive and curative traditional medicine preparations for human beings and livestock since ancient times [8]. The phenolics and flavonoids of medicinal plants contribute to the antioxidant activities of plants [9], thereby reducing the generation of free radicals [10] and alleviating diseases caused by oxidative stress [11].

*Croton macrostachyus* Hochst.ex Delile is a species of the genus *Croton* L., Euphorbiaceae family, commonly known as the spurge family [12]. It is a medium sized, drought-deciduous pioneer tree which regenerates naturally in less productive sites, including forest edges, mountain slopes, and waste grounds under a wide range of ecological conditions [13]. It has medicinal values, and is commonly used for the treatment of malaria, rabies, gonorrhea, diarrhea, hepatitis, jaundice, cancer, typhoid, pneumonia and gastrointestinal disorders and as ethno veterinary medicine [12, 14]. The genus Croton is rich in secondary metabolites with biological and/or pharmacological potential. Preceding studies exhibited the existence of crotin, lupeol, crotepoxide, proteins, fatty acids, saponins, resins and alkaloids. The action of *C. macrostachyus* root extracts is equivalent to studies where antioxidant, anti-bacterial, antiviral, anti-cancer activities has been associated to a variety of numerous classes of secondary plant metabolites including alkaloids and sesquiterpenes, triterpenes, flavonoids, inonoids, and quassinoids [15]. In Ethiopia, a few studies have been undertaken, which mainly emphasized on the determination of total polyphenols, flavonoids, antioxidant and antibacterial activities of *C. macrostachyus* leaves [16]. However, papers on the above-mentioned parameters, especially on the total polyphenolic contents and antioxidant activities, in the root samples of the plant is currently fragmentary and rare. Therefore, this study aimed at determining the total phenolics, flavonoids, antioxidant activity and antibacterial activities of *C. macrostachyus* root extracts.

## Materials and methods

### Study area description

**Fig. 1:**
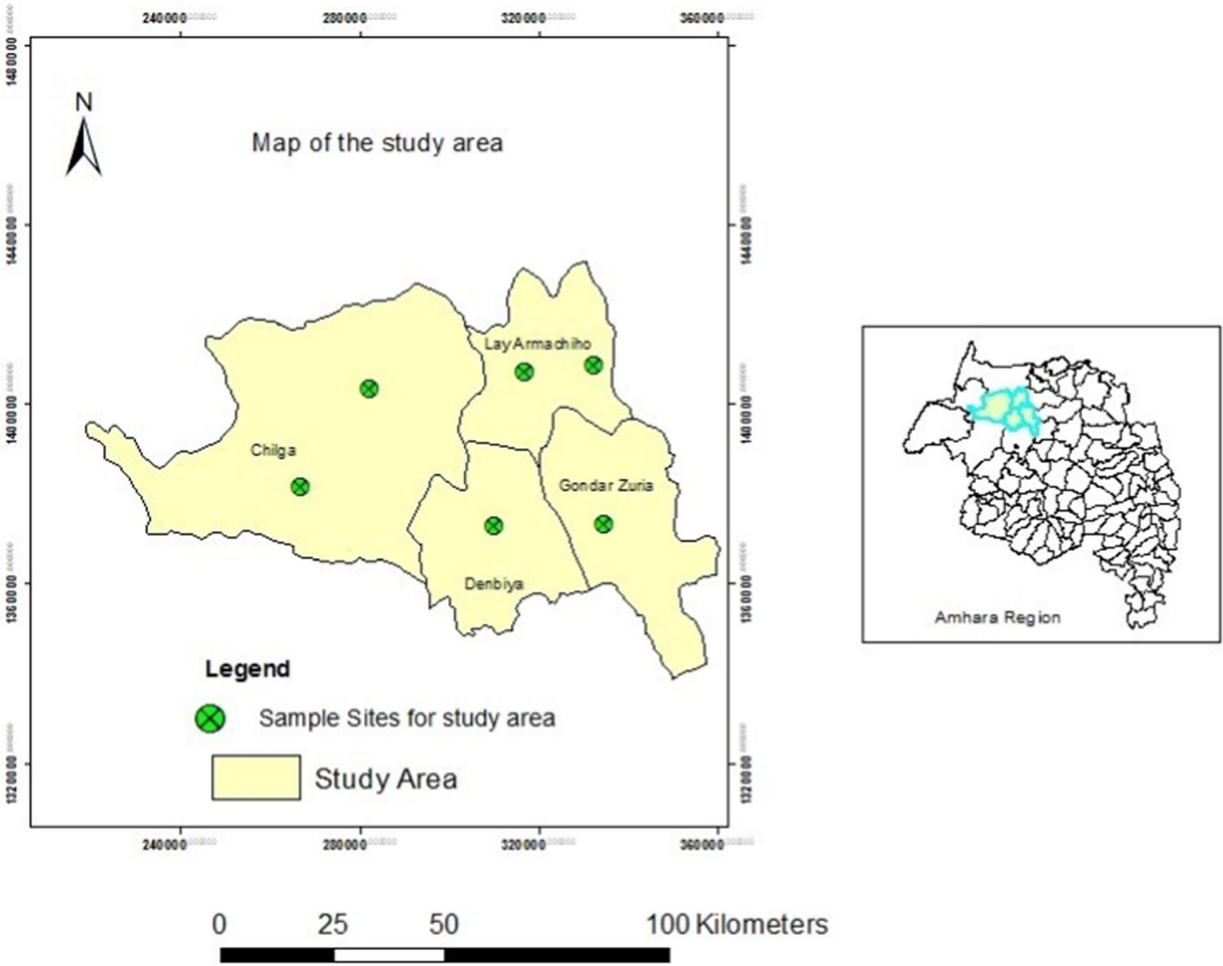
Map of study area (Sources: Wuletaw Mulualem)

The study was conducted on selected traditional plants collected from Chiliga, Lay Armachiho, Gondar Zuria and Dembiya Woredas, which ware situated in the Central Gondar Zone of the Amhara regional state (Fig. 1). The elevation of the Woredas ranged from 900 to 2267 m a.s.l. The temperature of the Woredas ranged from 11 to 32 °C with average annual rainfall of 995–1175 mm. The income of the local people is mainly based on sustenance mixed agriculture (crop-livestock production). The study areas were selected based on the large abundance of the plant and dependence of majority of its population on such traditional medicinal plants; and high prevalence of patients infected with rabies who took the root extracts of the plant as traditional medicine could be another reason to mention (Table 1).

**Table 1:** Geographical location of the sample areas

Sampling sites	Longitude	Latitude
S1	28^o^35′40″ E	14^o^03′146″ N
S2	26°64′34″ E	13°82′619″ N
S3	31°60′41″ E	14°08′705″ N
S4	33°18′64″ E	14°09′133″ N
S5	33°48′57″ E	13^o^73′639″ N
S6	31°04′81″ E	13^o^72′783″ N

### Chemicals and reagents

All the chemicals and reagents used in the present study were of analytical grade. The chemicals and reagents included: Methanol (98%), Folin-Ciocalteu reagent, gallic acid, ascorbic acid, sodium hydroxide, anhydrous sodium carbonate and anhydrous aluminum chloride, which were purchased from Loba Chemie (Mumbai, India); whereas 2,2-diphenyl-1-picrylhydrazyl (DPPH), sodium nitrite and catechin were purchased from Sisco Research Laboratories (Mumbai, India).

### Apparatus and equipments

Electrical grinder (IAK–WERKE, Germany), electronic balance (Bosch, Germany), Whatman No. 42 filter paper (110 mm), graduated pipettes, micropipettes (Mumbai, India), refrigerator (Hitachi LR902T, USA), orbital shaker (GEMMY Orbital Shaker, VRN-480, Taiwan), lyophilizer (Scientz-10N, Ningbo Scientz Biotechnology Co., Ltd., China), rotary evaporator (Bibby RE200, Sterilin Ltd., UK) and UV/Vis spectrophotometer (Sanyo SP75, UK) were used in the study.

### Collection of plant material, drying and storage

Identification and authentication of the plant was performed by a Botanist using taxonomic keys and floras, and voucher specimen (002/ATM/2020) was kept at Botanical Science and Herbarium, University of Gondar, for future references. Representative samples of *C. macrostachyus* root were collected from six sites. Permissions were obtained during plant material collection. From each site, 3 sampling points were selected; two aged plants were selected per sampling point, making a total of six plants per sampling site. Generally, a total of 36 matured plants were selected from 6 sites; and two root samples were collected per plant. Samples were collected from June to September, 2021 (wet season) and January–April, 2021 (dry season) in Ethiopian context. Plant root samples collected from each sampling site were homogenized to get a composite sample after removing surface contaminants by first washing with tap water, followed by distilled water. Here, the root samples were first dried at ambient temperature for three weeks under a plastic cover to avoid dust contamination, powdered using an electrical grinder, sieved through a 100-μm pore size sieve, then homogenized, and kept in clean polyethylene plastic bags until extraction.

### Optimization of extraction conditions

Phenolic contents and antioxidant activity were affected by solvent type, pH level, solvent–water ratio and extraction time [11]. In the present study, solvents ratio (methanol/water) and extraction time were optimized. Accordingly, the solvents ratio was optimized at ambient temperature by changing the methanol/water (v/v) ratio from 50 to 100%, while keeping the extraction time at 24 h. Likewise, the extraction time was optimized by varying the time from 12 to 72 h, while upholding the optimized solvent ratio (80%; v/v) at room temperature. In all cases, 0.5 g of the plant root powder was extracted with 25 mL aqueous methanol.

### Root extracts preparation

The dried and ground powdered root sample (0.5 g) was extracted with 25 mL of 80% aqueous methanol (v/v) by maceration using an orbital shaker at room temperature for 24 h. Then, the extract was filtered through Whatman No. 42 filter paper, and its volume adjusted with 80% methanol. Finally, the filtrate was stored in a refrigerator at 4 °C until further analysis. Extraction was performed in triplicate for each bulk sample and a reagent blank under the same conditions. However, for analysis of antibacterial activities, the methanol extracts were first condensed with a rotary evaporator, whereas the aqueous extracts were lyophilized. The dried extracts were stored with sterilized, sealed-culture tubes at 4 °C until each crude extract was re-constituted with 1 mL of distilled water.

### Preparation of standard solutions

Gallic acid standard was prepared by dissolving gallic acid in 80% methanol; 7.5% Na_2_CO_3_, 5% NaNO_2_, 10% ACl_3,_ 1 M NaOH solutions were prepared with double distilled water. Catechin and ascorbic acid standard solutions were also prepared with methanol.

### Total polyphenolic content

Total phenolic concentration in the root extract was spectrophotometrically determined by the Folin–Ciocalteu assay, using gallic acid as a standard [17, 18]. The reaction mixture was prepared by mixing 0.5 mL of plant root extract, 3 mL of distilled water and 0.25 mL of Folin-Ciocalteu reagent and shaken. After 5 min in the dark, 1 mL of 7.5% Na_2_CO_3_ was added and incubated at room temperature for 90 min in the dark. Reagent blank was also parallelly prepared using distilled water. The absorbance was measured against the prepared reagent blank at 760 nm using a double beam UV/Vis spectrophotometer. The concentration of total phenolic compounds in the extract was expressed as milligram of gallic acid equivalent (GAE) per 100 g sample (mg GAE/100 g). All the samples were analyzed in triplicate.

### Determination of flavonoid content

Flavonoid content was determined using aluminum chloride assay according to [18]. Briefly, an aliquot (0.5 mL) of the extract was added to a 10 mL test tube containing 2 mL of distilled water. To each test tube, 0.15 mL of 5% NaNO_2_ was added. After 5 min of incubation, 0.15 mL 10% AlCl_3_ was added. After 1 min, 1 mL of 1 M NaOH was added and the volume was adjusted to 5 mL with distilled water. After 10 min, the absorbance of the resulting solution was measured at 510 nm. Catechin was used as standard to express total flavonoids contents of samples as mg catechin equivalent per 100 g of sample (mg CE/100 g sample). All the samples were analyzed in triplicate.

### Determination of antioxidant activity

The antioxidant activity of the plant root extracts was evaluated using the DPPH method as described by [19] with slight modification. A mass of 20 mg of DPPH was dissolved with a small amount of methanol in a 500 mL volumetric flask. After the DPPH was fully dissolved, the flask was filled up to the mark with methanol. The control was prepared by mixing 3 mL of methanol and 2 mL of DPPH solution. In addition, a stock solution of ascorbic acid was prepared by dissolving 50 mg of ascorbic acid in 100 mL of methanol. Generation of calibration curve was achieved by preparing different concentrations, viz*.* 1, 2.5, 5, 10, 15, 20, 25, 50, 100, 150 and 200 mg/L, from the stock solution. To each test tube, 3 mL of standard ascorbic acid and 2 mL of DPPH solutions were added; then the test tubes were covered by aluminum foil and kept in the dark for 30 min. For the samples, a 0.1, 0.2, 0.4, 0.8, 1.6 and 2.4-mL portions of the extracts were mixed with 1.6 mL of DPPH and the final volume of each solution was adjusted to 4 mL with 80% aqueous methanol. The mixture was kept in the dark for 30 min. Finally, the absorbance was measured at 517 nm. The percentage of DPPH radical scavenging activity was determined using the formula:

$$\% {\text{ Inhibition}} = \left[ {{{\left( {{\text{A}}_{{{\text{DPPH}}}} - {\text{A}}_{{{\text{Sample}}}} } \right)} \mathord{\left/ {\vphantom {{\left( {{\text{A}}_{{{\text{DPPH}}}} - {\text{A}}_{{{\text{Sample}}}} } \right)} {{\text{A}}_{{{\text{DPPH}}}} }}} \right. \kern-\nulldelimiterspace} {{\text{A}}_{{{\text{DPPH}}}} }}} \right] \times 100.$$

where A_DPPH_ was absorbance of DPPH control solution and A_Sample_ was absorbance of DPPH solution in the presence of plant extract. Sample concentration giving 50% inhibition was estimated as IC_50_ value using the dose inhibition curve in linear range by plotting the extract concentration versus the corresponding scavenging activity. Measurements were performed in triplicates. The results were expressed as mg ascorbic acid equivalent per gram of sample (mg AAE/g sample).

### Evaluation of the antibacterial activity

The disk diffusion method was used to evaluate antimicrobial activity of the plant root extracts against four bacterial strains, namely: gram-positive bacteria species (*Staphylococcus aurous* and *Staphylococcus pneumonia*) and gram-negative bacteria species (*Escherichia coli* and *Klebsiella pneumonia)* using Gentamicin disc as standard drug [6]. These microorganisms were cultured at the molecular biology laboratory, Department of Biology, University of Gondar.

The media were prepared according to the manufacturer’s instruction as follows: 38 g of the Muller Hinton agar powder was dissolved in 1000 mL of distilled water, then heated, shaken well and allowed to boil and completely dissolved. Then, it was placed in an autoclave at 15 Pa pressure (121 °C) for about 15 min to sterilize the media. After that, the media were cooled, poured into 12 plates and put on a leveled surface. Lastly, the media were allowed to solidify, and kept in a laminar air flow hood in an upright position to avoid contamination. Lastly, the test culture bacteria were swabbed on the top of the pre-leveled media and allowed to dry. A sterilized Pasteur pipette was used to bore holes on plates and 100 μL of each extract was applied to the holes in triplicate using Gentamicin drug as positive control. The petri dish was incubated at 37 °C for 24 h. At the end of incubation period, the antibacterial activities of root extracts were determined by measuring the average inhibition zones of the extract and positive control in radius millimeter.

### Statistical analysis

All statistical analyses were undertaken using SPSS Version 20. Differences at p < 0.05 were significant.

## Results and discussion

### Effects of extraction conditions

Phenolic contents and antioxidant activity were affected by the type of solvent, pH level, solvent–water ratio and extraction time [20]. In the present study, the extraction efficiency of phenolic compounds was optimized as a function of the solvent/water ratio. First, the effects of extraction time and solvent ratio on the resulting extraction yield were determined by keeping one of the two extraction factors constant and varying the other. Throughout the optimization process, solvent composition (MeOH:H_2_O; v/v) had higher effect on the extraction efficiency, due to the difference in interactions of functional groups in plant materials with the solvent used [21].

**Fig. 2:**
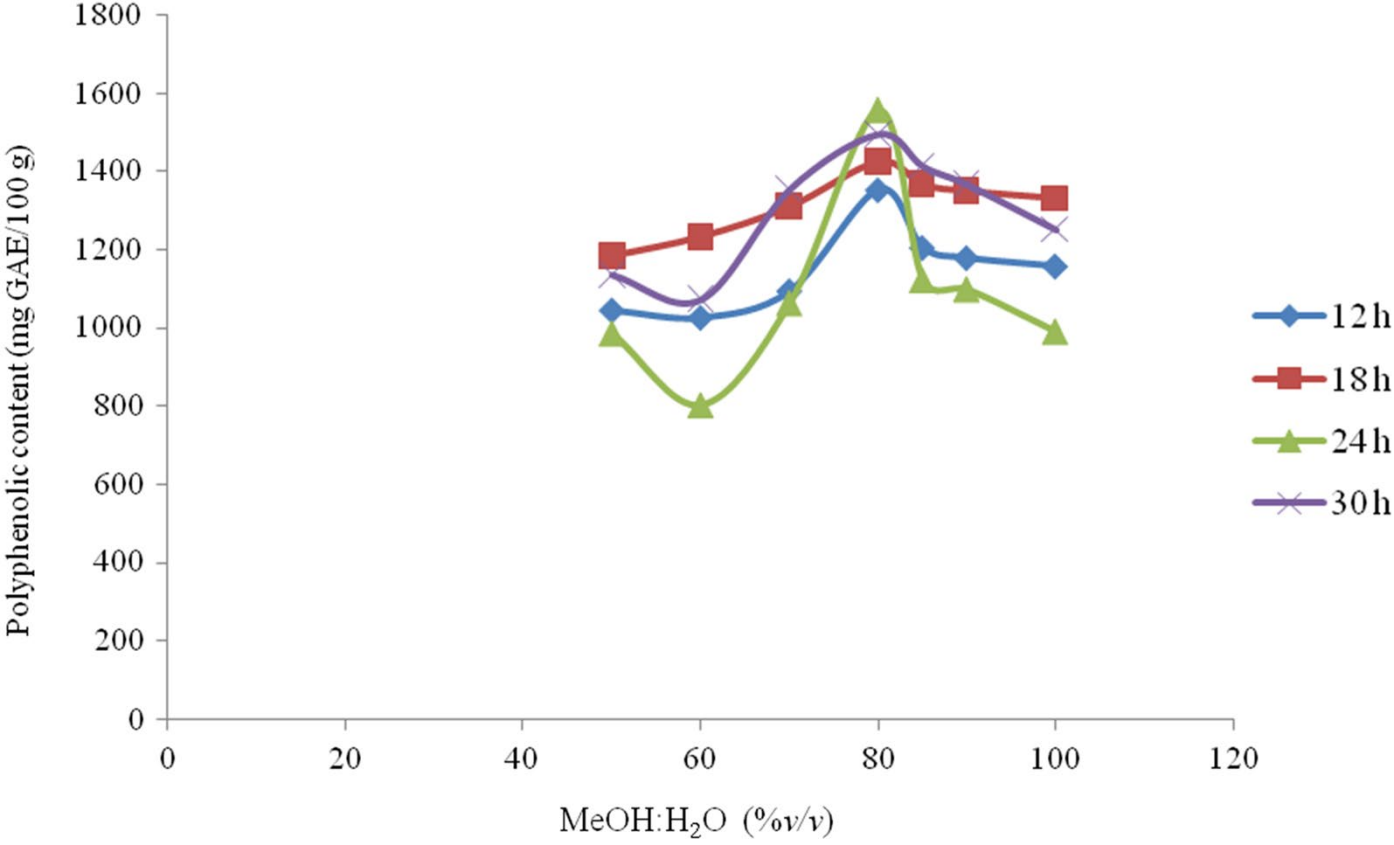
MeOH:H_2_O (%v/v) and extraction time (h) on the extraction efficiency of TPP from Croton macrostachyus: mass of plant root sample (0.5 g); volume of solvent (25 mL; MeOH:H_2_O); and at room temperature

As shown in Fig. 2, the concentration of total polyphenols differed significantly depending on the solvent ratio. Maximum extraction yield was obtained for a solvent with higher methanol ratio in comparison to lower ones. Varying the solvent ratio generally affected the extraction yield of polyphenols from the plant roots with the addition of 80% aqueous methanol being the optimal extraction solvent ratio; but the extraction efficiency started to slowly decline at higher methanol levels (> 80%).

Extraction time has also significant effect on extraction of total polyphenolic contents from the plant root extracts [22]. The present study showed that concentration of total polyphenols first increased, but started to decrease with time, attaining the optimal extraction time at 24 h (Fig. 2). The decrease in total polyphenolic contents at prolonged extraction times (> 24 h) could be due to spontaneous oxidation of some phenolic substances [23, 24]. In conclusion, 80% aqueous methanol (v/v) and extraction time of 24 h were selected as optimal conditions to extract 0.5 g root samples at ambient temperature.

### Total polyphenolic content

Phenolic compounds are important plant constituents with redox properties, thus imparting antioxidant activity since hydroxyl groups in these compounds are responsible for facilitating free radical scavenging [25]. They are believed to account for a major portion of the antioxidant capacity in many plants [26].

The total polyphenolic content varied from 802 ± 53 to 1557 ± 75 mg GAE/100 g of sample across the different sampling sites (Table 2). The mean polyphenolic content of root samples was 1105 ± 204 mg GAE/100 g of sample. Results obtained in this study showed that root samples collected from S2 and S5 had higher total polyphenolic contents, while lower values were recorded for samples collected from S4. These variations might be related to geographic and environmental factors. According to Ref. [27], natural extracts with proven antioxidant activity usually contain compounds with a phenolic moiety like flavonoids, carotenoids, tannins. Therefore, phenolic components are among the compounds responsible for reducing the DPPH radical.

**Table 2:** Total polyphenols (TPP) of *Croton macrostachyus* (mean ± SD, n = 3)

Sampling site	TPP (mg GAE/100 g) sample
S1	986 ± 98
S2	1557 ± 75
S3	1063 ± 6
S4	802 ± 53
S5	1122 ± 98
S6	1099 ± 86

The mean total polyphenolic contents of *C. macrostachyus* root extracts obtained in the present study was lower than other medicinal plants recorded in Indonesia [28], Serbia [29] and Italy [30], but were higher than those recorded for Ethiopia [31], Sri Lanka [32] and India [33]. The observed significant differences in the polyphenolic contents of various medicinal plants could be ascribed to such factors as plant root variety, growth conditions, climate, processing and analytical methods used [34]. The higher level of polyphenols recorded in the present study rationalizes the widespread use of the plant root in innumerable traditional herbal medications.

### Flavonoid content

Flavonoids are among the major groups of phenolic compounds [35], with broad spectrum of chemical and biological activities, particularly radical scavenging and antimicrobial activities [36]. They ubiquitously occur in plants and bring about beneficial health effects. Studies on flavonoid derivatives have revealed antibacterial, antiviral, anti-inflammatory, anticancer, and anti-allergic activities [37]. In this study, the flavonoid contents showed the same trend as that of the values recorded for total polyphenols. The mean concentrations of flavonoids in the *C. macrostachyus* root extracts ranged from 342 ± 26 to 745 ± 32 mg CE/100 g sample (Table 3)*.*

**Table 3:** The mean flavonoids contents of *Croton macrostachyus* (Mean ± SD, n = 3)

Sampling site	Flavnoid (mg CE/100 g) sample
Eyaho Seraba	489 ± 13
Teber Serako	745 ± 32
Aykel Town	470 ± 19
Laza Buladigie	342 ± 26
Bezaho Mekenet	495 ± 19
Nara Awurarda	476 ± 32

Among the collected root samples, the highest concentration of flavonoids was found in S2 site, whereas the lowest was found at S4. However, samples from the remaining sites had relatively similar flavonoid concentrations. As most abundant naturally occurring polyphenols in plants, flavonoids certainly provide protection against oxidative stress along with other oxidative defenses such as vitamins and enzymes [30].

### Antioxidant activities

Antioxidant activity of plants is largely attributed to the presence of bioactive compounds in them. This may not only be due to high percentage of main constituents, but also the presence of other constituents in small quantities [11]. In this study, the antioxidant activities of *C. macrostachyus* root extracts were determined by DPPH radical scavenging assay using ascorbic acid as a reference antioxidant standard. The values are expressed as milligrams of ascorbic acid equivalents per gram sample (mg AAE/g).

**Fig. 3:**
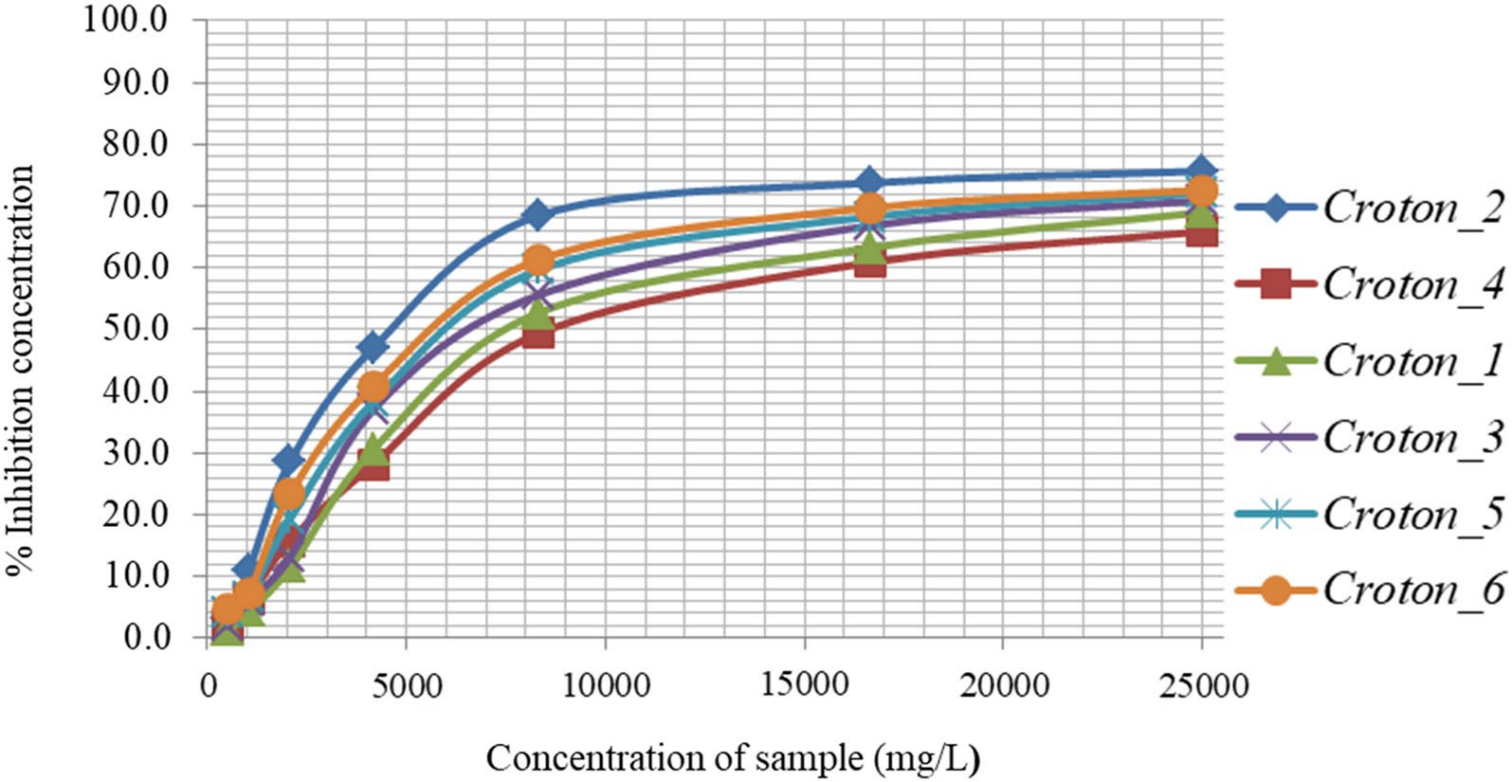
Inhibition of methanol water [80:20 (v/v)] extracts of *Croton macrostachyus* (Bisana)

The scavenging activities of root extracts that corresponds to their concentrations, inhibiting 50% of the DPPH radicals (IC_50_) are presented in Fig. 3. The calculated IC_50_ values for the root extracts were found in the range of 3.5–6.4 mg AAE/g of extract (Fig. 3), indicating good antioxidant potential of the plant root extracts. In the present study, the average antioxidant activities of the root extracts (4.8 mg AAE/g of sample) were smaller than those recorded in Indonesia [31], Serbia [32, 38], India [36]. The variation might be ascribed to the difference in phytogeographic region and plant nutrition, which could modify the secondary metabolites of the plant [31], as well as solvent polarity and extraction method differences [39]. The antioxidant activity of the root extracts and their respective total phenolic contents were positively correlated (r = 0.996, P < 0.05). The results suggested that the phenolic compounds contributed significantly to the antioxidant capacity of medicinal plants.

### Antibacterial activity

**Fig. 4:**
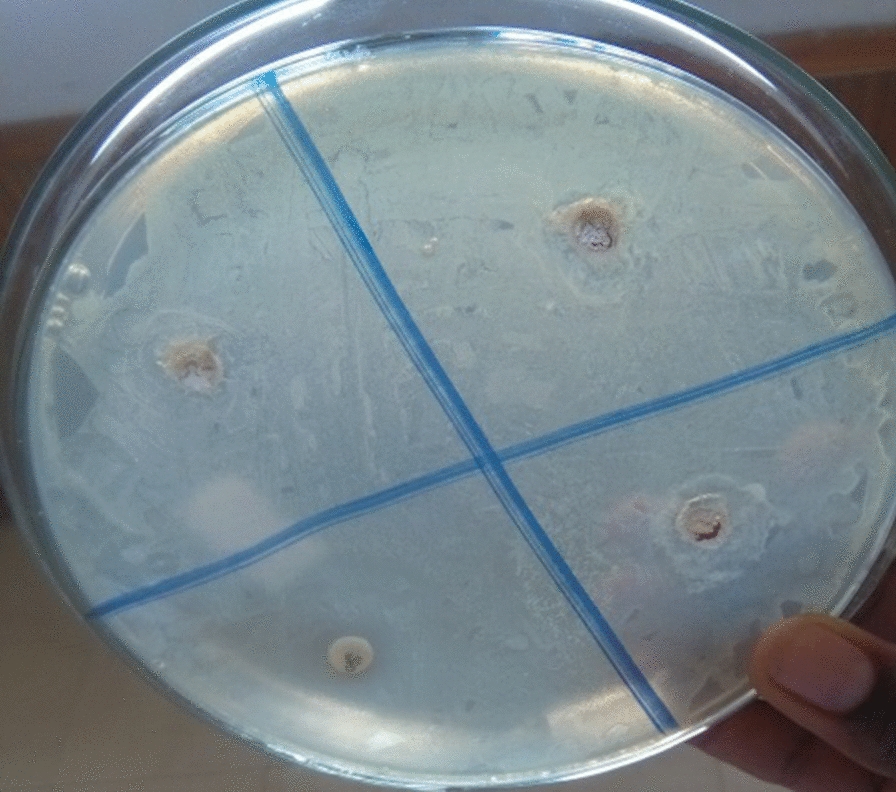
Zone of inhibitions of extracts in comparison with Gentamicin (positive control) against the four bacteria: *Staphylococcus aurous*, *Staphylococcus pneumonia*, *Escherichia coli* and *Klebsiella pneumonia*; for *Croton macrostachyus* plant extracts

Antibacterial components are used to cure diseases caused by bacteria. Hence, they appear to be good choice since artificial drugs have become resistant to many transmittable microorganisms [40]. In this respect, several studies showed that medicinal plants have bioactive components, like flavonoids and phenols, with such various functions as bacteriostatic, bactericidal, chemotherapeutic, and antimicrobial functions [40]. In this study, the antibacterial activity of methanolic crude root extracts were estimated using the disc diffusion method against gram- positive (*S. aurous* and *S. pneumonia*) and gram-negative (*E. coli* and *K. pneumonia*) bacteria that are common etiology of skin and wound infections. The findings revealed that *C. macrostachyus* root extracts showed inhibition zones against *S. aurous* (5.8 mm), *S. pneumonia* (6.2 mm), *E. coli* (5.9 mm) and *K. pneumonia (*6.0 mm) (Fig. 4). The observed high antibacterial activities of the root extracts, especially against *S. pneumonia* and *K. pneumonia,* is indicative of their potential use in promoting wound healing [41].

## Conclusion

In the present study, it was found that *C. macrostachyus* root extracts contained higher amounts of total polyphenols and exhibited higher antioxidant and antibacterial activities, making it a potential candidate for the treatment of bacterial-induced diseases. As a result, further study on the isolation of bioactive components from this plant root is in progress in our laboratory*.* Similarly, in vivo studies on its antibacterial activity using animal models is highly recommended to verify their practical use.

## Data Availability

All data generated and analyzed are included within this research article.
